# Case Series: Dual-Energy CT in Extra-Articular Manifestations of Gout: Main Teaching Point: Dual-energy CT is a valuable asset in the detection of extra-articular manifestations of gout

**DOI:** 10.5334/jbsr.2113

**Published:** 2020-06-03

**Authors:** Nando De Vulder, Min Chen, Wouter Huysse, Nele Herregods, Koenraad Verstraete, Lennart Jans

**Affiliations:** 1 Ghent University, BE; 2 Ghent University Hospital, BE

**Keywords:** Gout, Dual-Energy CT, Achilles Tendon, Tophus, Monosodium Urate Crystals

## Abstract

Extra-articular manifestations of gout are common. The Achilles tendon is a frequently affected site, and uric acid deposition may have harmful effects on tendon structure and function. Advanced imaging can aid in early diagnosis, follow-up of disease activity and therapy efficacy. This case series highlights the use of dual-energy CT as a tool in diagnosing gout and in detecting extra-articular manifestations.

## Introduction

Gout is the most common cause of inflammatory arthritis. It causes progressive joint destruction and may lead to monosodium urate (MSU) crystal deposition in soft tissues [1,2]. Usually, diagnosis is clear when patients present with typical signs and symptoms of gout. Nevertheless, atypical cases exist and may confront clinicians with diagnostic difficulties [3,4]. In this case series four patients are presented in whom gout was suspected and dual-energy computed tomography (DECT) was used to confirm or exclude the diagnosis.

## Case 1

A 65-year-old man presented with chronic inflammatory pain in the 1st metatarsophalangeal joint (MTP- I) of his right foot. Clinical examination showed no gout tophi. Blood uric acid was 6.1 mg/dL (normal <7 mg/dL). CT images were obtained using a dedicated DECT scan protocol for gout, scanning at tube voltages of 140 kV and 80 kV. Data sets were processed with SyngoVia (Version VB20, Siemens Healthineers, Erlangen, Germany) and a gout-specific algorithm that colour-coded MSU crystals in green.

**Figure 1:**
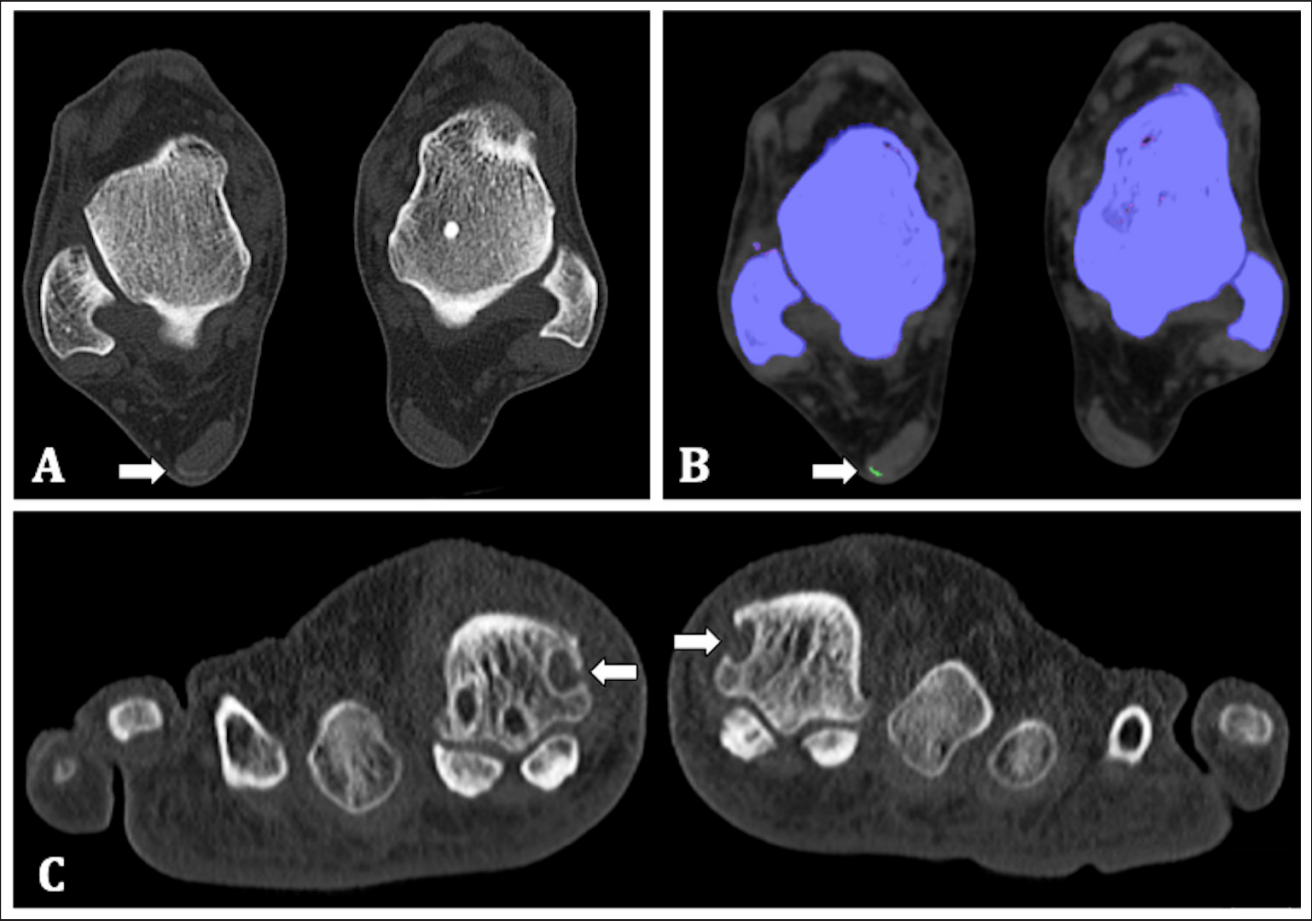
**65-year-old man. (A)** Transverse native CT image of both feet at the level of the talus. A higher-density zone is shown in the lateral margin of the Achilles tendon (arrow). **(B)** Colour-coded images identified the presence of MSU crystals as a green zone (arrow). **(C)** Coronal native CT image, showing erosions with sclerotic border and overhanging edges in the head of the first metatarsal bone both left and right (arrows).

The native CT images showed erosions with a sclerotic border and overhanging edges in the MTP-I of both feet and a higher-density zone in the lateral margin of the Achilles tendon of his right foot with MSU crystal deposition on DECT, all typical features seen in gout [5] (Figure 1).

## Case 2

A 50-year-old man consulted because of ongoing pain in his right Achilles tendon, painful recurrent swelling in both ankles for the last two years and one episode of joint pain at the MTP-I several months earlier. Clinical examination showed no signs of local inflammation or tophus formation. Blood uric acid was 7.4 mg/dL. The same DECT setup as in the first case was used.

**Figure 2:**
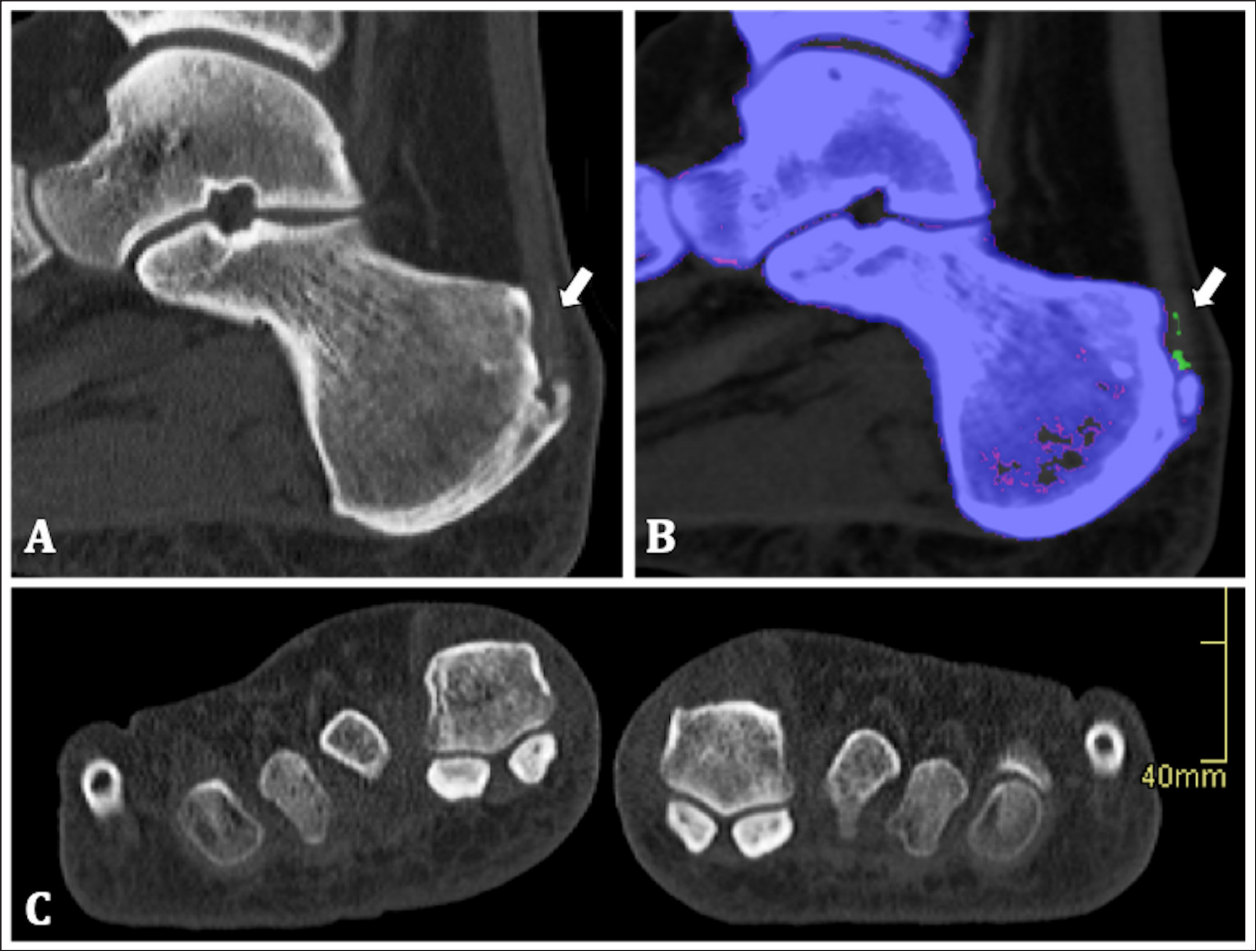
**50-year-old man. (A)** Sagittal native CT image of the right calcaneum. Calcaneal enthesophyte and several linear areas of high density in the Achilles enthesis are shown (arrow). **(B)** Colour-coding identifies the linear densities as uric acid crystals (arrow). **(C)** Coronal native CT image shows there were no erosions of the MTP-I.

Native CT images showed calcaneal enthesophytes and high-density zones in the right Achilles tendon at the insertion with the absorption spectrum of MSU crystals. The patient had no joint degeneration or erosions in neither of the feet (Figure 2). Nevertheless, the patient could be diagnosed with gout and MSU crystal deposition in the Achilles tendon.

## Case 3

A 70-year-old woman presented with persisting pain in both ankles and the left Achilles tendon for two years. Recently the pain also started in her right Achilles tendon and the MTP-I on the left. She had pressure pain in both ankles, a painful swelling of the right Achilles tendon and 4.7 mg/dL uric acid in the blood. DECT was performed to help differentiate between gout and calcific tendinopathy. The same DECT setup as in the previous cases was used.

**Figure 3:**
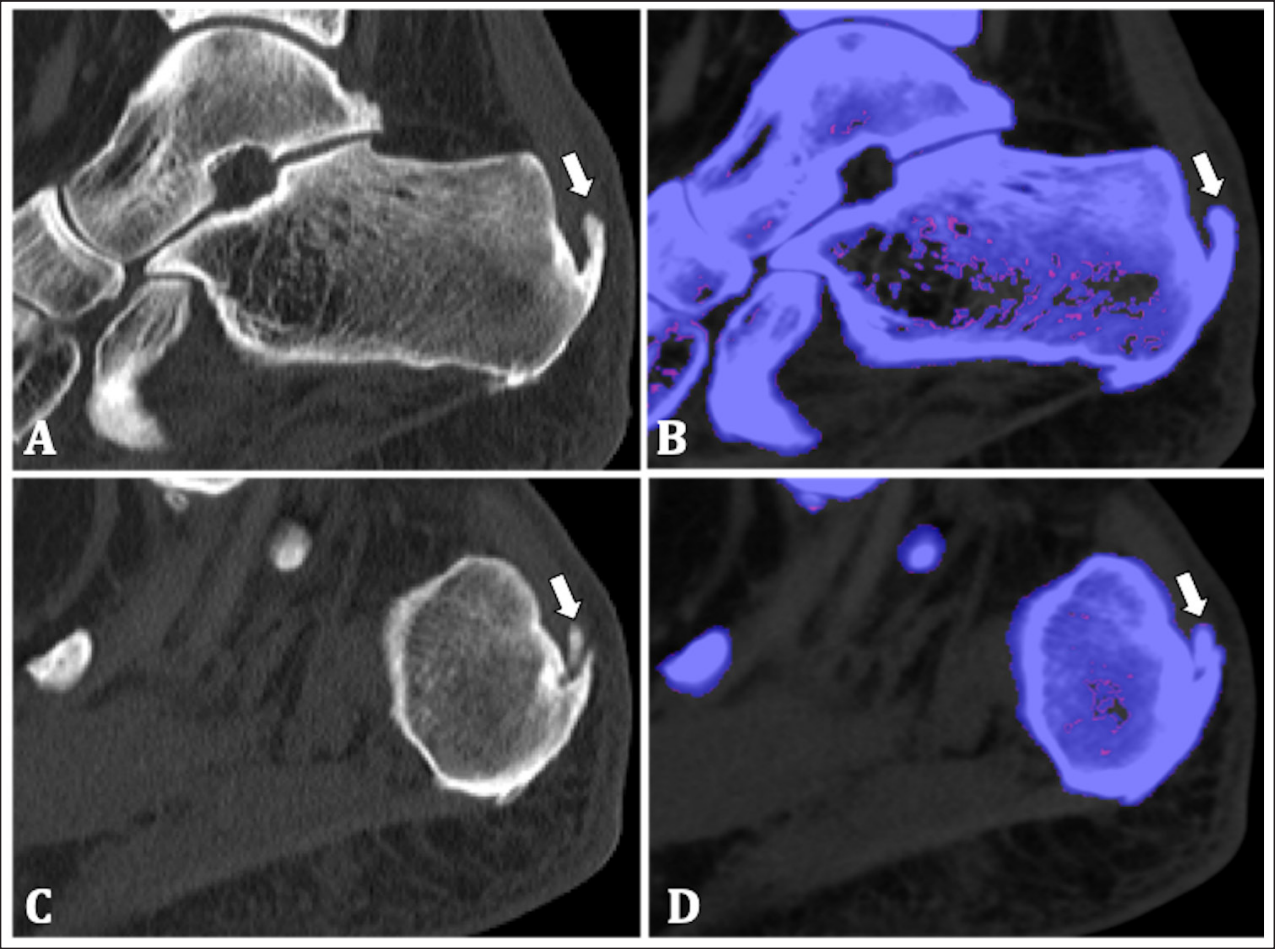
**70-year-old woman. (A, C)** Sagittal native CT image of the left and right calcaneum respectively show enthesophytes at the Achilles tendon insertions (arrows). **(B, D)** Post-processing shows there is no uric acid deposition (arrows).

DECT showed enthesophytes at the insertion of the left and right Achilles tendon and bilateral calcaneal spur, most dominantly on the left. No erosions or MSU depositions were found (Figure 3). These findings suggested a mechanical etiology of the patient’s articular pain and gout was excluded.

## Case 4

A 70-year-old man presented with painful swelling of the right Achilles tendon. Three months prior to presentation he had experienced pain at the MTP-I of his right foot. He had a history of right Achilles tendon rupture, surgically corrected. Clinically, there was mild pressure pain at the right Achilles tendon, starting at the enthesis and spreading towards the middle third of the tendon. There were no clinically apparent tophi. Blood uric acid was 7.4 mg/dL.

**Figure 4:**
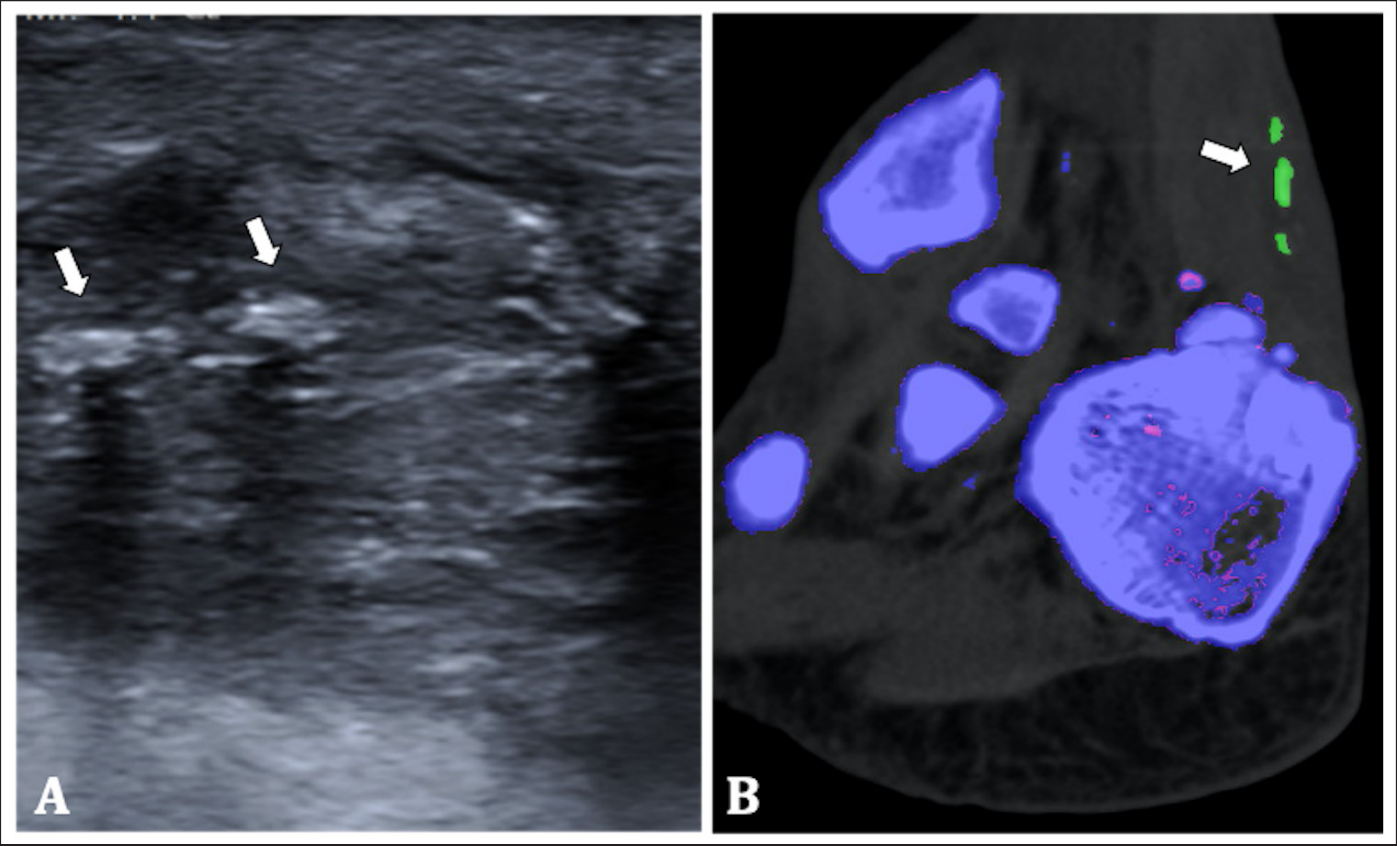
**70-year-old man. (A)** Ultrasound of the right Achilles tendon shows tendon thickening and multiple hyperechoic nodules (arrows). **(B)** Colour-coded DECT image shows MSU crystal deposition in the Achilles tendon (arrow).

Ultrasound (US) of the tendon showed signs of inflammation and multiple hyperechoic nodules (Figure 4). To differentiate between tendinous calcifications or MSU crystal depositions DECT was performed. The same setup as in the previous cases was used. Colour-coded DECT images showed the presence of MSU crystals in the right Achilles tendon (Figure 4). Therefore, the patient was diagnosed with oligoarticular gout with tendinous involvement.

## Discussion

The cases above demonstrate dual-energy CT as a helpful tool in detecting extra-articular MSU depositions, both in typical and atypical cases. DECT can differentiate MSU crystals from hydroxyapatite depositions in calcific tendinosis or osteophytosis [6,7] and prove essential when US fails to aid in diagnosis.

Joint or tophus aspiration remains the golden standard in establishing the diagnosis of gout. However, sufficient aspiration is not always possible and requires a trained clinician [8,9]. This is where non-invasive imaging modalities like US and DECT can be particularly helpful. DECT combines the imaging properties of conventional CT imaging, with the possibility of processing the images to identify MSU crystal depositions [4]. US accuracy is operator dependent and DECT has proven to be superior to US for identifying total urate deposition and assessing volume of deposits [10,11,12].

The Achilles tendon is one of the most common locations of extra-articular tophus manifestation [13]. It has been demonstrated that MSU crystals exert an inhibitory effect on collagen production and induce tenocyte apoptosis. This could disrupt tendon structure and self-repair. Detection of tendon involvement in gout is important because it could lead to tendon damage in people with advanced gout [14]. Therefore, DECT can be particularly helpful in identifying these sites of uric acid deposition, even when they are clinically not apparent [7].

DECT can also serve as an important diagnostic aid in cases of painful tendon swelling or pseudotumoral involvement of tendons. This way DECT can help in the atypical presentation of gout when solely extra-articular tophus formation is found devoid of any signs of arthritis [3].

## Conclusion

DECT is a valuable asset in the diagnosis of gout, especially when extra-articular involvement is present. MSU crystal deposition in tendons may affect tendon structure and therefore functionality. DECT can help evaluate total uric acid crystal load and has proven to be superior in this assessment than other non-invasive methods.
